# Synthetic AI-Generated Satellite Imagery to Improve Earth Observation-Based Neural Networks

**DOI:** 10.3390/s26123895

**Published:** 2026-06-18

**Authors:** Enrique Albalate-Prieto, Noelia Vallez, José Luis Espinosa-Aranda, Aubrey Dunne, Raúl Barba-Rojas

**Affiliations:** 1 Ubotica Technologies, DCU Alpha, Old Finglas Road 11, Glasnevin, D11KXN4 Dublin, Ireland; raul.barba@ubotica.com; 2 E.T.S. Ingeniería Industrial, Avda, Camilo José Cela, S/N, 13071 Ciudad Real, Spain; noelia.vallez@uclm.es

**Keywords:** data augmentation, generative AI, earth observation, remote sensing

## Abstract

Recent advances in satellite technology have significantly progressed, yet acquiring high-quality images with meaningful labels for Earth observation missions remains a costly and time-intensive process. Furthermore, captured scenes frequently exhibit defects such as misaligned color channels, extensive cloud cover, or repetitive patterns in similar environments. Fortunately, the evolution of generative artificial intelligence offers a solution by enabling the creation of realistic synthetic scenes, simulating the characteristics of any targeted imager, and thereby mitigating the scarcity of authentic data. This paper demonstrates the feasibility of transferring knowledge from specialized AI-generated datasets to Earth observation missions. Leveraging a novel dataset of Spanish map tiles, Pix2Pix, CUT, and ControlNet models were implemented to synthesize satellite imagery. To analyze structural and topological generalizability, identical U-Net instances were trained on the resulting collections for building, road, and water segmentation tasks, and subsequently tested on independent authentic imagery. The results reveal a clear decoupling between visual realism and functional utility. Incorporating synthetic samples into hybridized training datasets successfully surpassed the limitations of using real data alone, increasing maximum Dice scores by 0.9% (to 54.1% for buildings), 2.3% (to 38.6% for roads), and 4.1% (to 46.5% for waterbodies). This systematic validation establishes structural-guided synthetic data augmentation as a robust, adaptable strategy for Earth observation applications across diverse sensors and geometric objectives.

## 1. Introduction

Earth Observation (EO), the process of gathering information about our planet using remote sensing technologies, has been established as a fundamental tool for global monitoring. This continuous perspective of the Earth’s surface becomes feasible primarily as a result of the information provided by satellites, especially their resulting imagery. These scenes, combined with machine learning techniques, can be used for a wide range of tasks, including the tracking of illegal human activities (e.g., illicit constructions [1], deforestation [2], or unregulated mining [3]), the early detection of natural disasters such as floods or wildfires [4], and the control of maritime traffic and border security [5].

Despite the increasing availability of sensors, the development of robust and high-performance computer vision systems for spaceborne applications still faces a persistent challenge: the scarcity and low quality of labeled datasets [6,7]. Real EO images often exhibit problems inherent to real-world acquisition, such as cloud occlusion and band misalignment (which invalidate large portions of the data), or repetitive patterns when taken from geographically similar areas. These deficiencies, coupled with a costly and time-consuming extraction process, have led to a lack of scenes acquired with specific sensors [8,9].

In this context, the development of artificial-intelligence-based generative models has gained attention as a research path to overcome these issues [10,11]. This approach entails the creation of high-fidelity synthetic samples whose content can be tailored and controlled as needed, resulting in a promising data augmentation strategy to construct specialized datasets for any desired purpose. Consequently, training neural networks on synthetic scenes resembling a particular sensor’s characteristics has emerged as a popular alternative to automate and conduct detection missions [12,13].

**Figure 1:**
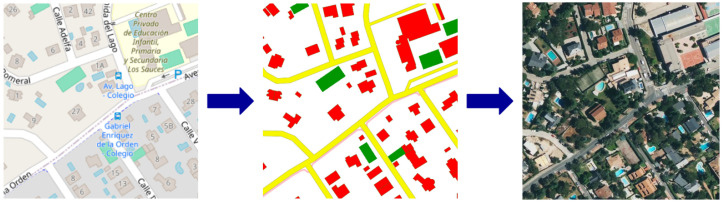
Evolution of input data from left to right: a raw map crop, its formatted version, and an example of a synthetic satellite image created via the generative architectures used in this work.

This study primarily aims to investigate the feasibility of transferring knowledge applicable in real-world scenarios to neural models relying on artificial images created by certain manageable cutting-edge generative architectures. As specified in Figure 1, the experiments were concentrated on acquiring computer-synthesized satellite imagery by feeding the networks with abundant and readily available formatted map tiles, and evaluating their usefulness through semantic segmentation tests over authentic EO data. Significantly, while the previous literature often heavily relies on subjective human perception or isolated tasks [10,12,13], this work addresses a critical research gap. Specifically, it systematically analyzes how structural alignment and texture synthesis impact downstream task accuracy across three structurally and topologically diverse entities: buildings, roads, and waterbodies. The findings prove the benefits of combining real and synthetic samples to enhance the performance achieved solely with the former. Furthermore, this framework supports the utility of AI-generated satellite data for future applications aimed at reducing the labeled real imagery requirements and mitigating model overfitting. Finally, the current limitations of this data augmentation technique are identified by revealing a distinct decoupling between visual photorealism and functional semantic utility.

The rest of this document is structured as follows. Section 2 begins by reviewing the foundational concepts and latest advancements in classical and state-of-the-art approaches of image generation paradigms. Section 3 details the comprehensive methodology, including the specific datasets, procedures, and techniques employed throughout this study. The presentation and analysis of the quantitative and qualitative results are covered in Section 4. Section 5 discusses the main limitations encountered and proposes directions for future research. Finally, Section 6 offers a brief summary of the completed research and highlights its core contributions.

## 2. Research Background

Image generation intrinsically differs from classic computer vision problems due to its fundamentally unsupervised nature. While standard tasks like classification or regression learn to predict known outputs, image generation needs to model the data’s underlying distribution without relying on explicit labels, developing the ability to produce samples visually similar to the training examples. Early attempts at synthetic scene creation focused on texture models and statistical methods. These techniques aimed to replicate local image properties by means of histograms or spatial correlations, resulting in a successful reproduction of homogeneous patterns but failing to construct globally coherent structures [14,15]. Subsequently, the first approaches based on neural networks, such as the Restricted Boltzmann Machines and Deep Belief Networks, were introduced in this field. These models sought to generate novel images by learning hierarchical latent representations and abstract input data features [16,17]. Nevertheless, their training was often computationally expensive and their scalability was very limited.

To introduce a differentiable probabilistic formulation, Variational Autoencoders (VAEs) were subsequently proposed for this task. The significance of VAEs lies in their capacity to simultaneously address three core issues: learning a continuous latent space, allowing meaningful interpolations between encoded images, and facilitating the generation of new scenes by sampling from a structured prior distribution [18,19]. The VAE architecture is fundamentally composed of two main components: an encoder, which maps the input data to a latent space, and a decoder, which reconstructs the image from that latent representation. Although this proposal could also be properly applied to data compression [20] and anomaly detection [21], the primary limitation of VAEs is their tendency to produce blurry images [18,22,23].

Alternatively, Generative Adversarial Networks (GANs) represented a paradigm shift in the image synthesis field by establishing a zero-sum (minimax) game between two neural networks: a generator and a discriminator [22]. The generator aims to create scenes so realistic that they can deceive the discriminator, while the latter seeks to effectively distinguish between the real data samples and the fake ones produced by the generator. Both components, with opposing learning objectives, mutually improve through an iterative competition process. This strategy refined the quality of the artificial outputs achieved by those adversarial models. GANs stood out for their notable behavior in several generative missions such as image-to-image translation, style transfer, and domain adaptation. However, they commonly suffer from training instability (difficulty in balancing the adversarial competition) and model collapse [24]. This latter phenomenon restricts the diversity of their outputs, which is a critical limitation when constructing valid augmentation datasets that improve models’ generalization.

In order to overcome the inherent limitations of traditional GANs, conditional architectures such as Pix2Pix were introduced [25]. These models, instead of starting from pure noise, utilize a complementary input image as guidance, allowing the model to learn a deterministic mapping between pairs of images: the auxiliary input and the generated output. This supervised schema partially stabilizes the training process and enables the creation of results consistent with the input. However, this method relies heavily on strictly paired datasets, which are commonly difficult and expensive to obtain in many contexts.

With the aim of eliminating this data restriction, CycleGAN incorporated the innovative cycle-consistency loss [26]. This mechanism allows the network to learn relationships between domains without requiring aligned pairs. By utilizing two generators and two discriminators that operate in opposite directions (from domain A to domain B and vice versa), the architecture ensures that a translated image preserves its initial identity by subsequently being reconverted to its original domain. This breakthrough significantly expanded the applicability of conditional GANs to unpaired scenarios. Nonetheless, it introduced a considerable computational cost and a strong dependency on satisfying the mentioned cycle consistency constraint, occasionally resulting in less natural outputs or visual artifacts.

In response to this complexity, the Contrastive Unpaired Translation (CUT) architecture proposed replacing the cycle loss with a contrastive loss computed between internal representations [27]. This successfully achieved unpaired translation between domains using only a single generator and a single discriminator. This efficiency is attained by focusing on shared domain features (e.g., shapes, object parts) while simultaneously remaining invariant to certain differences (e.g., textures). This simplification drastically reduced the complexity and training requirements, maintained competitive visual quality, and made cross-domain translation more accessible.

A distinct probabilistic approach emerged with the introduction of Denoising Diffusion Probabilistic Models (DDPMs) [28]. These models initiate their learning process during training by gradually introducing Gaussian noise to real images over multiple steps, continuing until the scenes are entirely transformed into pure noise. The network is then trained to learn the reverse operation: progressively removing that noise to reconstruct the original image. Ultimately, DDPMs manage to synthesize artificial samples starting solely from random noise, refining them step-by-step. In light of the explicitly probabilistic nature of this method, DDPMs succeeded in capturing exceptionally fine visual details and achieving high-fidelity results, which have usually been shown to be more coherent and stable [29,30].

Modern generative frameworks frequently combine the structural strengths of these distinct paradigms through hybrid formulations. A particularly popular example of this is the combination of VAEs and DDPMs, the Latent Diffusion Model (LDM) approach [23]. It consists of a diffusion process applied within compressed latent spaces to reduce computational costs and offer more flexible control over the generated content. In addition, conditional control mechanisms have been integrated into large models, for instance, through text embeddings (e.g., Imagen [31], or DALL·E 2 [32]) or architecture augmentations with trainable networks to enforce spatial or structural constraints (e.g., ControlNet [33]), enabling the generation to be guided by precise semantic instructions.

These last massive solutions effectively mitigate the limitations of previous stages: they improve fidelity compared to VAEs, stabilize training against GANs, and accelerate inference compared to basic DDPMs. In contrast, many recent works focus on more affordable alternatives given that the computational resources necessary to train or deploy these foundational models are prohibitive for many applications. Authors in [34] provide a comprehensive survey of efficiency-oriented techniques for diffusion models, reviewing algorithmic and architectural strategies designed to reduce the cost of training and preserve generative capabilities. Moreover, in [35] authors propose a lightweight GAN-based architecture for high-dynamic-range image reconstruction that relies on depthwise separable convolutions to significantly reduce parameters and FLOPs while maintaining competitive visual quality.

## 3. Materials and Methods

This section establishes the experimental workflow designed to evaluate and compare the utility of synthetic imagery in remote sensing operations. The methodology encompasses two core phases: the implementation of structurally diverse generative paradigms and the subsequent evaluation of their synthesized datasets via downstream semantic segmentation models.

### 3.1. Image Generation Methods

Throughout this research, multiple generative paradigms were evaluated: from conditional and contrastive learning-based GANs to a cutting-edge LDM that emerged from the hybridization of a DDPM and a VAE, together with the application of prediction control mechanisms like ControlNet.

The selection of these generative architectures was guided by distinct computational and structural criteria. First, given that the image generation task is computationally intensive, special consideration was given to the model size and execution time requirements of the potential candidates. Furthermore, the primary goal of this study was to assess the feasibility of employing synthetic satellite images to train models that would subsequently perform automatic EO tasks on authentic scenes captured with specific sensors. Bearing this in mind, models that are distinct from one another in terms of implementation strategy were prioritized to determine which training and generation approach is more suitable. Specifically, the following image-to-image translation approaches were compared:

- Pix2Pix, a model built upon the framework of conditional Generative Adversarial Networks [25]. Its primary goal is to learn a deterministic mapping between two strictly aligned visual domains, exploiting the pixel-wise correspondence between input and target images. As a result, both the generator and the discriminator are conditioned on the input image, which constrains the solution space and encourages structural consistency in the generated outputs.
  The generator follows a U-Net architecture, consisting of an encoder that extracts hierarchical representations from the input image and a decoder that reconstructs the target image. The discriminator adopts the PatchGAN approach, assessing the likelihood of small local image patches rather than the entire image, which encourages the synthesis of realistic textures while reducing the number of trainable parameters. In addition, this model is optimized using a composite loss function that combines an adversarial objective with a pixel-wise reconstruction loss, which penalizes direct deviations from the target image.
  In terms of the network implementation, its PyTorch (v2.6.0) version [36] was preferred to the model’s original code in the Lua language (Torch) [37]. The primary architectural discrepancy is that the Lua framework utilizes a 7-layer encoder (instead of 8) along with a symmetric 7-layer decoder. The rest of the details from the official paper were maintained.
- CUT, an architecture designed to work without requiring the target domains to contain aligned image pairs [27]. CUT introduces a contrastive loss that operates on the internal representations of the generator, promoting the preservation of structural content between the input image and its translated counterpart. This allows the model to avoid enforcing explicit reversibility constraints from the target domain back to the source domain.
  Accordingly, the model employs a single generator and a single discriminator, which significantly reduces computational complexity compared to other cycle-consistency-based approaches. The core of this approach lies in patch-wise contrastive learning: the similarity between features extracted from each region of an input image and the respective region in the generated image is maximized, while similarity with features from other spatial locations is minimized. This mechanism compels the generator to maintain semantic and geometric correspondences between domains even with substantial appearance differences.
  In this case, the original implementation of the neural network [38] allows training under both unpaired and paired data loading strategies, making it possible to conduct objective comparisons of their impact on final architectural performance. It is essential to note that, although the CUT model was designed to operate with randomly sampled images from each domain (maps and satellite scenes), the unpaired methodology underscores two notable limitations in the context of this study. Firstly, random sampling does not guarantee full dataset coverage within a single training epoch. Secondly, it was initially conceived for translation between highly similar domains, such as horse-to-zebra translation, whereas the map-to-satellite translation task considered here involves large differences in visual detail and semantic complexity.
- ControlNet, an extension of diffusion-based models that introduces explicit mechanisms for structural control during the generation process [33]. Diffusion models formulate image generation as a probabilistic process that progressively inverts a sequence of Gaussian noise degradation steps, which results in training stability and a comprehensive coverage of the target data distribution.
  The ControlNet architecture is based on the duplication of a pre-trained U-Net: one branch remains frozen, preserving the knowledge already acquired, while the other is trained to incorporate external control signals, such as maps, edges, or spatial structures (e.g., sketches). Both branches are connected through zero-initialized 1 × 1 convolutions, which ensure that control information is integrated in a gradual and stable manner without degrading the original generative behavior.
  To make this approach computationally feasible, the diffusion process is performed in a compressed latent space following the Latent Diffusion Model paradigm [23]. In this setting, images are projected into a lower-dimensional latent space using a separately trained VAE, and diffusion is applied over these representations. Beyond the substantial reduction in terms of computational costs, this mechanism leads to a precise control over the spatial structure of the generated outputs without compromising visual quality in conditional translation tasks.
  Due to hardware constraints, the implementation selected for this study focused on a faithful yet lighter approximation of the original framework [39], utilizing a smaller U-Net backbone. This architecture enables the gradual integration of map-based control signals and latent representations processing, while maintaining the probabilistic advantages of DDPMs in capturing fine-grained details.

### 3.2. Dataset

**Figure 2:**
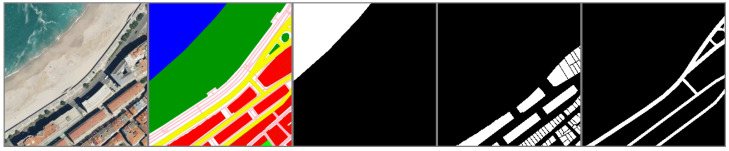
Examples of binary masks extracted through color filtering applied to the dataset maps.

The primary objective of dataset curation was to facilitate the synthesis of high-quality satellite images from accessible map tiles using neural networks, providing a scalable alternative to limited data sources. Furthermore, given the absence of any appropriate existing dataset, Mapbox [40] was chosen as the cartographic data provider to address the map-to-satellite translation task. As a result of its control over style, scale and zoom level, it is well-suited for creating training datasets composed of accurately correlated image pairs combining map crops and their corresponding satellite scene. This platform enables extensive map customization, allowing distinct cartographic features to be rendered in specific, high-contrast colors (see Table 1) for two important reasons. Firstly, sketches representing structures using contrasting colors have been commonly utilized to improve generative architectures’ predictions [41,42]. Secondly, this modification enabled the automatic extraction of binary masks focused on any desired entity by filtering their associated color (consult Figure 2), facilitating future semantic segmentation experiments.

MapBox provides a global imagery layer by integrating multiple satellite and aerial sources to deliver contextual and high-quality backdrops for maps. According to the official documentation, the imagery’s sensors vary based on the zoom level. Since we set it to 17, the satellite data was extracted from Maxar’s Vivid basemap, which provides consistent, cloud-free mosaics with a typical Ground Sample Distance (GSD) ranging between 30 cm and 50 cm per pixel depending on the region. In some areas, Mapbox also supplements this with open context-rich national aerial datasets.

**Figure 3:**
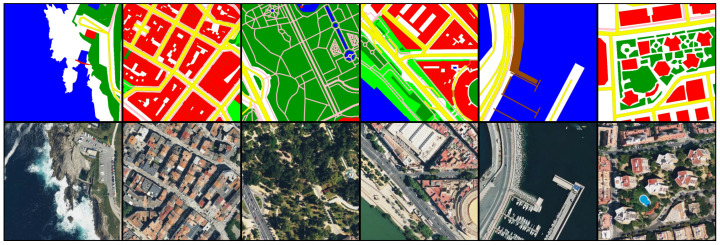
Examples of dataset items: formatted maps and their corresponding satellite images.

A varied dataset was initially created to make generative models capable of properly managing any input map once trained. Given that a wide range of environments was needed (e.g., city centres, roads and highways, forests, crops, mountains, coasts and harbours), Spain was selected as the target location to focus on during this phase. The process proposed to create the custom dataset started by computing different landmarks belonging to the Spanish boundary silhouette. This was achieved through multiple polygons constructed using the coordinates of some desired places, from which random points were sampled. Thus, those landmarks were employed to send requests to the MapBox API (Application Programming Interface) [43], which allows users to download both map tiles and their associated real satellite images, as can be seen in Figure 3. Moreover, after a dataset curation step, all pairs comprising (i) censored satellite images taken from government and military facilities or (ii) uniform map crops without enough meaningful details (belonging to the cities’ outskirts) were discarded. Finally, a training set (5276 items) and a test set (587 items) were obtained. In contrast with discriminative approaches, generative ones such as GANs and diffusion models do not rely on a validation set for model selection, since their training objective does not optimize for a supervised generalization metric. Instead, the generator is trained to match the data distribution of a desired domain, and stopping criteria are typically based on fixed training schedules or qualitative inspection [22,28]. For this reason, only a held-out test split was used to visualize qualitative performance on unseen samples, which is consistent with standard practice in the generative modelling literature.

**Figure 4:**
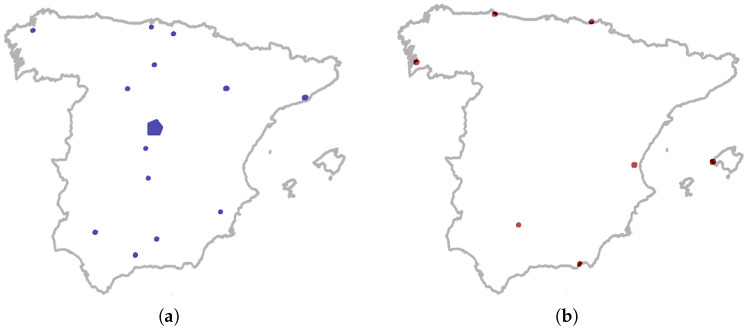
(**a**) Spanish regions chosen to train the generative architectures. (**b**) Spanish regions selected to evaluate the methods for generating synthetic satellite scenes.

A second dataset was created to assess, through semantic segmentations, the productions of the previously trained generative models. The new set of images required throughout this evaluation phase was acquired following the same process introduced before. Nevertheless, the random groups of landmarks were placed at different Spanish cities, as shown in Figure 4. Maps were extracted from the mainland and the Balearic Islands. This way, high diversity of data was guaranteed while also increasing the difficulty for the generative neural networks, which had to manage novel regions they had not seen during training.

In contrast to the generative architectures, a validation split was required for the segmentation framework. Also, instead of relying on map crops, they were replaced with binary masks automatically extracted from them by color filtering, to facilitate the detection of specific land-cover elements in any input satellite image (see Figure 2). As a result, a new dataset composed of 3574 filtered image pairs (satellite scenes and their corresponding masks) was obtained. To characterize the class distribution and inherent spatial density of the primary targeted features, the pixel-level labeling proportions were calculated, yielding 18.70% for buildings, 9.93% for roads, and 4.04% for waterbodies. Finally, the collection was partitioned into three independent subsets: training (80%, 2859 items), validation (10%, 357 items), and test (10%, 358 items).

### 3.3. Evaluation

**Figure 5:**
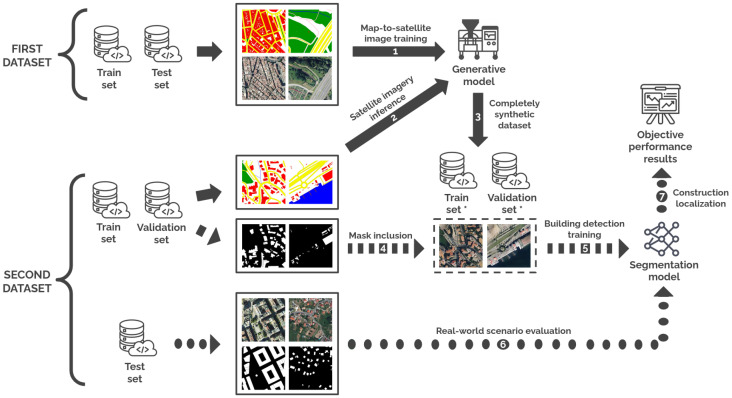
General pipeline for assessing knowledge transfer from synthetic satellite data to real-world sensor imagery, exemplified through the building segmentation task.

To derive objective conclusions, a comprehensive evaluation pipeline was followed. Given that the underlying methodology remains completely identical regardless of the target land-cover element, this global workflow is exemplified in Figure 5 using the building segmentation task as a representative structural baseline. The process began with the construction of the described extensive and varied dataset, consisting of paired instances of real satellite images and their associated maps. Subsequently, the chosen generative architectures were trained, under uniform conditions, to improve their capacity to generate artificial satellite scenes. While the previous literature addresses the reverse process (map production from authentic satellite imagery) [25,44,45], this work focused on inferring finer details such as textures or shadows instead of creating uniform areas.

Moreover, quantitative evaluations of the acquired synthesized samples are often omitted in the generic image-to-image synthesis literature [46]. Nonetheless, this research relies on a multi-task semantic segmentation framework to conduct a rigorous, application-oriented assessment. The strategy was based on testing a group of identical instances of a segmentation model, each of them respectively trained on synthetic data produced by the generative networks under study, with the same validation collection of real satellite image crops. In this manner, this setup assesses the feasibility of knowledge transfer from one domain in which arbitrary amounts of customized examples are readily available (maps) to another that is much scarcer and less accessible (satellite imagery captured by a target sensor).

To validate the limits of this knowledge transfer, the evaluations were extended across three distinct geographic and topological categories, each presenting unique structural challenges for computer vision models:

- Buildings (Discrete Entities): Representing highly fragmented, man-made structures characterized by a massive diversity of shapes, sizes, textures, and sharp geometric boundaries, which traditionally introduce significant appearance challenges [47,48].
- Roads (Linear Networks): Representing continuous, narrow curvilinear features that require the generative models to preserve complex topological connectivity and long-range spatial dependencies over extensive landscapes [49,50].
- Waterbodies (Dense Fluid Masses): Representing high-density, irregular polygonal areas characterized by wide spectral variations, diffuse coastlines and severe occlusions, testing the networks’ capacity to synthesize homogeneous surface properties [51,52].

By analyzing this heterogeneous set, the benchmark provides a robust perspective on how synthetic data augmentation operates across different structural typologies.

To implement this multi-task setup, a new dataset composed of a separate set of maps and their associated authentic satellite images was acquired. Then, the trained generative models were executed on these previously unseen maps, thereby obtaining entirely synthetic evaluation collections. Thereafter, the automatic extraction of the ground-truth binary masks corresponding to buildings, roads, and waterbodies was accomplished via the presented straightforward color filtering step. Finally, the training of several copies of the same U-Net architecture was conducted on these artificial datasets and the original real one, enabling fair testing protocols to compare the quality of the artificial samples not only among themselves but also with respect to the real baseline data.

The selection of the U-Net architecture for all downstream semantic segmentation tasks is justified by its well-established status as a cornerstone framework for Earth observation and remote sensing applications [53,54]. Recent comparative analyses also show that U-Net-based approaches achieve competitive performance against other segmentation architectures across multiple datasets while maintaining a relatively low computational overhead [55]. These factors make it a suitable alternative in the context of on-board satellite integration and real-time inferences [56,57]. The adapted U-Net implementation [58] ensures consistency with the reference design of the architecture proposed by its original creators [59], featuring a symmetric encoder–decoder with skip connections while adding modern refinements like batch normalization, and bilinear upsampling.

To evaluate the outcomes of the segmentation tests, established and widely recognized performance metrics for semantic mapping tasks were utilized. Specifically, Precision, Recall, and the Dice score were selected to provide a balanced assessment [60,61]. Precision quantifies the reliability of positive spatial predictions, ensuring that predicted pixels correspond to the targeted feature. In contrast, Recall determines the proportion of ground-truth entities successfully identified by the model, regardless of the overall volume of the spatial predictions. The Dice score, which combines both cited metrics, offers a complete perspective of the networks’ performance. Furthermore, the Dice score penalizes both under- and over-segmentation, precisely representing the models’ overall semantic mapping capabilities.

### 3.4. Experiment Configuration

We avoided the use of pretrained weights to prevent potential biases related to the statistics and semantic distributions of the original training data, ensuring a fair comparison between the evaluated models [23,29]. All architectures were trained from scratch, following the methodology discussed throughout the prior sections. In addition, each model utilized the baseline hyperparameter configurations suggested by its respective foundational literature (see Section 3.1).

Regarding data preprocessing, the input map tiles fed into the generative models were normalized to the [−1.0,1.0] range using a mean of 0.5 and a standard deviation of 0.5 across all color channels. This preprocessing step strictly conformed to the default, optimal training settings established in the foundational literature for Pix2Pix [25], CUT [27], and ControlNet [33], ensuring maximum gradient stability during the adversarial and diffusion workflows.

To ensure full reproducibility, a fixed random seed of 1111 was set for all generative model experiments, while a seed of 1 was chosen for the U-Net architectures. Furthermore, no data augmentation techniques were applied to any of the models during training since the primary objective of this research was not to over-optimize individual network performance. Conversely, it sought to establish a strictly uniform and controlled baseline environment that allows for a direct comparison of the knowledge-transfer capabilities inherent to each generative approach.

Based on empirical observations, the training duration for all generative networks was unified at 400 epochs. Beyond this threshold, the visual quality and structural fidelity of the synthesized outputs demonstrated negligible improvements, indicating full convergence. Additionally, a particular detail common to all artificial satellite scene creation experiments was the use of 256 × 256 pixel RGB input images, coupled with a batch size of 1. This parameter selection was primarily dictated by hardware constraints: specifically, a workstation equipped with an AMD Ryzen 9 7900 CPU (12 cores), 16 GB of RAM, and an NVIDIA RTX 3090 GPU with 24 GB of VRAM. Under these VRAM limitations, preserving the maximum possible spatial resolution of the input data was prioritized over processing multiple samples per iteration. Support for this approach can also be found in the literature [25,26,27]. Among them, two are particularly relevant:

- Using batches of size 1 allows models to focus on the specific details of individual examples instead of averaging out the features contained in larger sets.
- The gradients derived from such small batches are significantly noisier than in a normal training, which can assist the network in escaping suboptimal solutions or regions (especially when dealing with heterogeneous datasets).

All downstream U-Net architectures trained to evaluate the quality of the synthetic scenes employed the same input size. However, they were trained for 200 epochs using a batch size of 8 samples. These configurations represented the maximum sustainable workload for the mentioned equipment available throughout the research.

Table 2 summarizes the most important hyperparameters utilized by the models selected in this study, while Equations (1)–(6) present the mathematical definitions of the loss functions used in their training. Here, the abbreviations BCE, MAE, and MSE denote Binary Cross-Entropy, Mean Absolute Error, and Mean Squared Error, respectively. To distinguish the functional role of each loss component, the subscript “*I*” (Identification) indicates that the loss function is applied to distinguish artificial images from real ones; the subscript “*R*” (Reconstruction) denotes that it is used to measure the disparities between authentic scenes and their corresponding synthetic counterparts; and “*S*” (Segmentation) states that it compares the binary masks predicted by a segmentation model after processing satellite crops against their corresponding ground-truth targets.

In Equations (1)–(5), $x_{i}$ refers to an individual pixel within the input scene (whether real or synthetic), $d_{i}$ represents the ground-truth domain label ($d_{i}=1$ for authentic scenes, $d_{i}=0$ for synthetic samples) used during discriminator inference, and ${\hat{d}}_{i}$ signifies the scalar prediction generated by the discriminator. Furthermore, $a_{i}$ denotes the *i*-th pixel of an authentic target satellite image, whereas ${\hat{a}}_{i}$ represents the corresponding synthesized pixel generated from its paired map tile. Lastly, $m_{i}$ denotes the ground-truth pixel label within the binary mask indicating building, road, or water elements, and ${\hat{m}}_{i}$ signifies the target pixel activation value predicted by the downstream U-Net segmentation framework. Let *N* denote the total number of pixels per image instance:

(1) $${\mathrm{BCE}}_{I}\left(x\right)=-\frac{1}{N}\sum_{i=1}^{N}\left[d_{i}\log\left({\hat{d}}_{i}\right)+\left(1-d_{i}\right)\log\left(1-{\hat{d}}_{i}\right)\right]$$

(2) $${\mathrm{MSE}}_{I}\left(x\right)=\frac{1}{N}\sum_{i=1}^{N}{\left(d_{i}-{\hat{d}}_{i}\right)}^{2}$$

(3) $${\mathrm{MSE}}_{R}\left(a\right)=\frac{1}{N}\sum_{i=1}^{N}{\left(a_{i}-{\hat{a}}_{i}\right)}^{2}$$

(4) $${\mathrm{MAE}}_{R}\left(a\right)=\frac{1}{N}\sum_{i=1}^{N}\left|a_{i}-{\hat{a}}_{i}\right|$$

(5) $${\mathrm{BCE}}_{S}\left(x\right)=-\frac{1}{N}\sum_{i=1}^{N}\left[m_{i}\log\left({\hat{m}}_{i}\right)+\left(1-m_{i}\right)\log\left(1-{\hat{m}}_{i}\right)\right]$$

Additionally, the Noise Contrastive Estimation (NCE) framework is integrated into Table 2. Its execution requires extracting localized spatial patches from both the generated output and its paired source map [27]. For each patch sampled from the artificially synthesized scene, its structurally aligned counterpart in the source image is defined as the positive sample, while all remaining mismatched patches from the same scene serve as negative instances. The NCE loss function forces the generator to maximize the similarity between positive patch embeddings while simultaneously minimizing mutual information for negative locations within the projected feature space. In Equation (6), *v* represents the feature embedding of a selected query patch from the generated image, $v^{+}$ denotes its corresponding positive target embedding from the source image, and $v_{n}^{-}$ represents the set of negative patch embeddings. The hyperparameter τ denotes a constant temperature scaling factor, strictly set to its default value of 0.07:

(6) $$\mathrm{NCE}\left(x\right)=-\log\frac{\exp(v\cdot v^{+}/\tau )}{\exp\left(v\cdot v^{+}/\tau \right)+\sum_{n=1}^{K}\exp\left(v\cdot v_{n}^{-}/\tau \right)}$$

## 4. Results

This section presents a comprehensive qualitative and quantitative evaluation of the synthesized satellite imagery. First, the visual fidelity of the scenes generated by the different architectural paradigms is examined. Subsequently, the functional utility of these synthetic images is evaluated through downstream semantic segmentation tasks to quantify the knowledge transfer they enable toward the domain of real EO.

### 4.1. Image Generation Results

Regarding the Pix2Pix outputs, the best inferences were obtained on map patches extracted from urban city centres because these zones have higher detail levels than non-urban areas and are updated more often. However, it is worth noting that, in general, the synthetic scenes produced by the Pix2Pix model suffered from severe blurring, resulting in entities or structures almost indistinguishable to the human observer. Besides, qualitative analysis indicates that the network showed difficulties with map tiles containing large water surfaces (regions colored in blue), leading to white spot artifacts in the generated satellite images.

In contrast, the realism of the generated scenes by both CUT instances, respectively trained following an unpaired and a paired approach, was much higher. This architecture not only managed to preserve the structures’ edges within the detailed urban city centre map tiles, but it was also able, in the majority of cases, to solve the issue related to wide areas covered by water. Nonetheless, several issues were detected, such as: significant noise introduced during inferences while processing big uniformly colored areas, confusion between crops and large constructions, or wrong color association to some parks and gardens.

**Figure 6:**
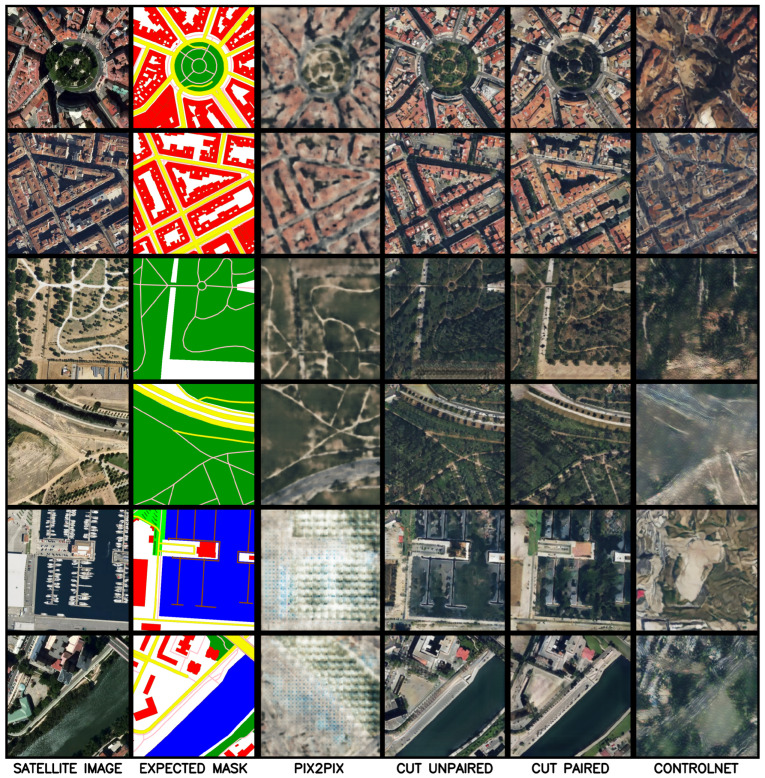
Examples of artificial images provided by the generative models used in this study.

Finally, the synthetic satellite images created by the ControlNet architecture exhibited noticeably low quality. This loss of detail may be related to the extreme dimensionality reduction of input real scenes when encoded into latents. Setting that assumption aside, no in-depth analysis could be conducted due to the limited visual quality of the outputs. In terms of urban city centres, ControlNet led to strange, unusual mosaics composed of geometric forms. Moreover, regarding other less populated zones containing water surfaces, industrial facilities or without any construction, the returned images were unintelligible: blurry color blends that seem to convey no usable information, at least from the point of view of a human observer. Figure 6 presents several outputs of the analyzed models, which illustrate the qualitative findings detailed within this subsection.

### 4.2. Semantic Segmentation Results

**Figure 7:**
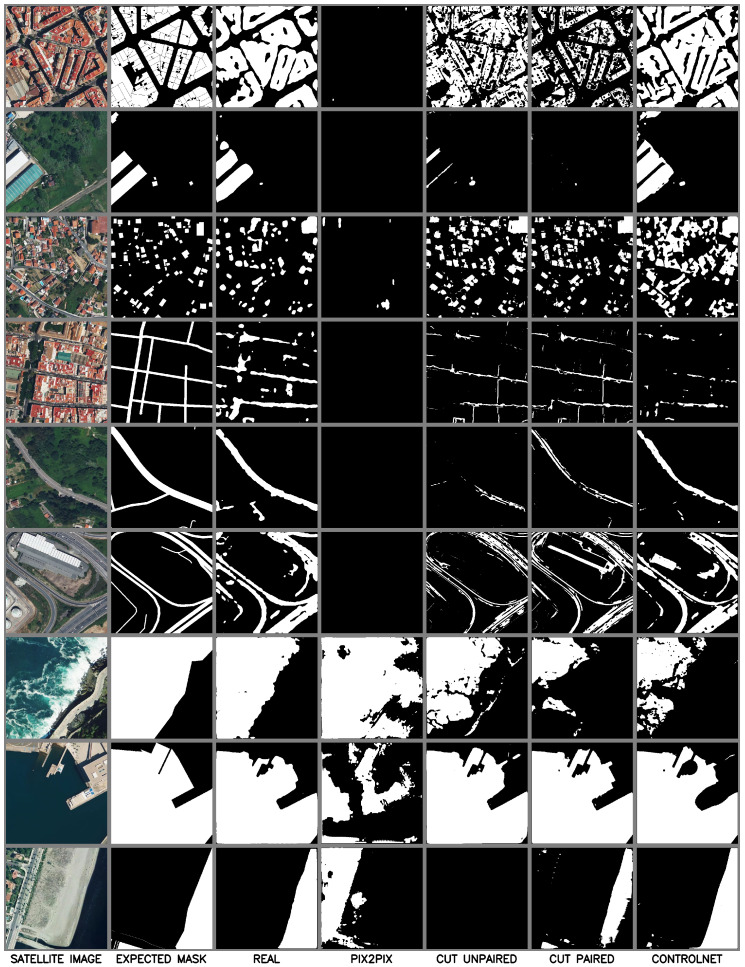
Examples of binary masks obtained using U-Nets trained on synthetic data from the chosen generative approaches.

To implement this evaluation setup, a U-Net instance was employed for each generative model obtained throughout the assessment phase of the project. Specifically, four segmentation networks were initially trained and validated on image sets consisting of synthetic satellite images respectively created by the Pix2Pix, CUT (unpaired and paired versions) and ControlNet architectures, as commented in Section 3.2. Therefore, all these learning processes were conducted without introducing any real scene so the knowledge acquired by the U-Nets could be consistently evaluated. In addition, a fifth U-Net was trained on the authentic satellite imagery the generative models should have imitated during the construction of the training and validation sets seen by their corresponding segmentation network. This model acted as the reference benchmark, defining the maximum achievable performance when working exclusively with real data, which comprised the final test set. Several conclusions can be extracted by reviewing the binary masks predicted by the U-Nets trained; thus, representative examples are provided in Figure 7.

Fundamentally, the structural blurriness characteristic of the Pix2Pix architecture prevented the U-Net trained on them from learning how to distinguish human constructions, roadways, and water masses from other elements. Consequently, while almost all pixels belonging to the authentic evaluation scenes were classified as ground for the first two objectives, the exact opposite failure mode was observed in the third task, where the model systematically over-segmented the images by predicting the targeted water features across nearly the entire layout.

Regarding the behavior of the U-Nets fed with CUT-generated synthetic scenes, these segmentation networks occasionally delineated well-defined boundaries in high-density urban areas. However, this localized visual sharpness did not translate into global segmentation precision. In particular, many false positives were observed when processing less populated areas. Crucially, while the U-Nets leveraging CUT-generated images succeeded in partially localizing a wide range of targeted entities, the primary drawback identified was that the resulting predicted masks were geometrically fragmented or incomplete, frequently failing to encapsulate the entire continuous layout of the recognized land-cover elements.

Despite the sub-optimal visual fidelity of the ControlNet outputs, counterintuitive binary masks were produced by the U-Net that employed them. Although a tendency towards false positives was discovered, the architecture demonstrated remarkable detection capacities. These findings suggested that the incomprehensible synthetic satellite images made by ControlNet still contained representative data, from the neural network perspective, for the segmentation task.

In light of the strengths and weaknesses of synthetic satellite images perceived in this first experiment, a second one was proposed in order to leverage the former and mitigate the latter. Thus, additional U-Net instances were trained after respectively combining the image sets from the two previously mentioned approaches (CUT paired and ControlNet) with the authentic pictures. As a result, the training and validation sets were composed of 5718 and 714 satellite images in each case (the original configuration was preserved).

**Figure 8:**
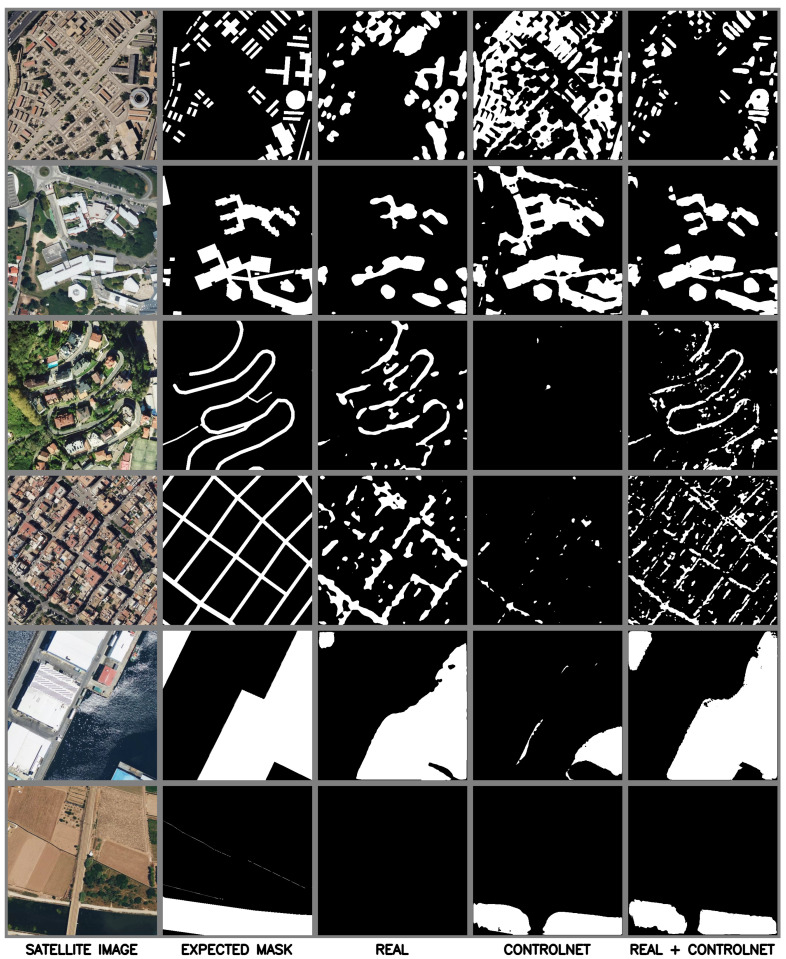
Representative examples showcasing the segmentation benefits of combining real and synthetic data derived from ControlNet in difficult scenarios.

The binary masks acquired from these hybrid experiments revealed that the mixed approach enhanced the predictions of the U-Nets solely trained on synthetic scenes as well as the baseline model trained on real imagery. The improvements yielded can be summarized as follows: (i) a refined management of large target entities and a reduction in land confusion; (ii) a higher geometric fidelity along structural edges. Furthermore, beyond all the advantages mentioned, the U-Net trained on ControlNet creations and real satellite scenes tended to produce competitive qualitative results for constructions, road networks, and water surfaces even when capturing irregular or uncommon shapes (see Figure 8).

Subsequently, a rigorous quantitative evaluation was performed with all the segmentation networks involved in the evaluation phase of this study. Nevertheless, it is essential to take into account the existing disparities between the input satellite images and the binary masks automatically extracted from their corresponding map tiles. This issue established a low feasible upper limit on the computed performance statistics due to the numerous cases in which accurately identified constructions, thoroughfares, or water surfaces were later considered as false positives. At any rate, Table 3 contains all the quantitative information related to the U-Nets’ performance when tested on real satellite imagery. Multiple conclusions were derived from these results:

1. Poor performance was observed for the segmentation models trained on the artificial imagery generated by the Pix2Pix architecture. The 3.2%, 2.0%, and 4.3% Dice scores made explicit the fact that the U-Net failed to learn meaningful representations across all targeted land-cover tasks.
2. For the U-Nets trained on images generated by the CUT neural network, no generation variant demonstrated a clear advantage over the others. While the paired strategy yielded better results for building and road segmentations (28.6% vs. 25.7% and 22.5% vs. 14.1%, respectively), the unpaired alternative achieved higher Dice score for waterbodies (40.0% vs. 31.5%). Furthermore, excluding the building task, the U-Nets leveraging these CUT-synthesized collections showed the highest performance scores among all segmentation models trained exclusively on synthetic data.
3. Although combining real scenes with those generated by the paired CUT network was insufficient to surpass the baseline model in building segmentation (48.4% vs. 53.2%), this hybrid approach matched the baseline performance for water masses (42.0% vs. 42.4%) and even outperformed it for road networks (38.0% vs. 36.3%).
4. Counterintuitive results were obtained after training a segmentation model with the blurry and non-photorealistic outputs of the ControlNet architecture, which yielded 44.4%, 16.7%, and 22.6% Dice scores. Despite the low visual quality of these images, the U-Net trained on this dataset exhibited generalization performance comparable to that of the models trained on data generated by the CUT architectures.
5. The mixed strategy combining real scenes with ControlNet-generated images consistently achieved the best performance across all three target classes. Specifically, it outperformed training on real data alone, increasing the maximum Dice scores from 53.2% to 54.1% for buildings, from 36.3% to 38.6% for roads, and from 42.4% to 46.5% for waterbodies.

The extracted statistics were then refined in order to gain a clearer understanding of the existing performance gap among the segmentation models depending on the biome of the input scenes. An empirical inspection of the map tiles associated with the satellite images belonging to the test set revealed that only two distinct environments could be automatically identified: (i) urban city centres with plenty of buildings; (ii) the remaining non-urban settings, involving unpopulated regions with no constructions such as forests, highways, and coasts, as well as industrial parks with remote large facilities.

**Figure 9:**
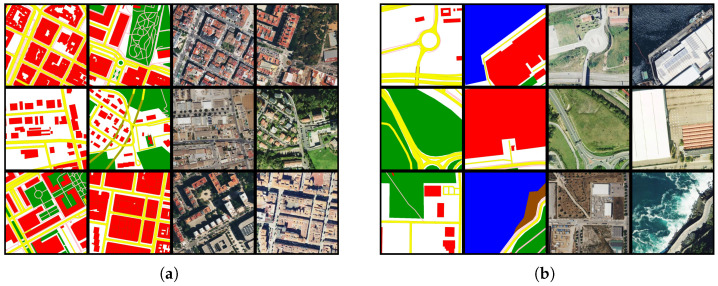
Illustrative image pairs automatically classified as city centres (**a**) or assigned to the other subset (**b**).

Keeping this in mind, three building-related features were examined to split the evaluation set into both groups: building size, total count, and area. These features defined an ad-hoc criterion to determine whether an analysed map tile, and its corresponding real satellite image, should be assigned to one subset or another. It was established that every city centre map tile should contain at least 15 buildings covering a minimum area of 15% of the overall area, with no individual construction occupying more than 20% of the image’s total size. Representative examples are presented in Figure 9.

After this classification, the behavior of the already trained U-Net models was evaluated again on these two new sets. Table 4, which contains the results of the described assessment, shows that the U-Net models achieved higher performance when conducting inferences on the urban city centres set (217 items) compared to non-urban scenes (141 items), specifically for the man-made structural tasks (i.e., buildings and roads). Conversely, for the water segmentation objective, the models frequently maintained competitive or superior baseline metrics in rural and natural landscapes, where large-scale natural waterbodies are typically situated. Consequently, the assumption that these neural networks better manage densely detailed topological maps was supported primarily for urbanized geometric features, while natural land-cover entities exhibited a more balanced spatial distribution across both subsets.

## 5. Discussion

Previous works on synthetic Earth observation imagery primarily assess the realism of generated scenes through visual inspection or perceptual metrics [10,12,13]. However, the ultimate usefulness of synthetic data lies in its ability to objectively improve other downstream tasks. In this work, the quality of generative models was therefore tested through a semantic segmentation benchmark, providing a more application-oriented evaluation.

Pivotally, the model producing the most visually convincing samples was not the one that yielded the best segmentation performance. While CUT-generated images seemed more realistic to human observers, the scenes created by ControlNet led to higher downstream accuracy when combined with real ones. This observation supports the idea that perceptual realism does not necessarily correlate with the statistical properties required for machine learning models, a phenomenon already discussed in early studies of texture perception and synthesis [14,15]. To assess whether this observation generalizes across distinct structural typologies, evaluations were systematically conducted on three separate tasks: building, road, and water segmentation. Despite ControlNet-generated scenes occasionally exhibiting localized visual blurring or non-photorealistic textures, the downstream U-Net models trained on these images achieved competitive generalization performance when tested on authentic samples for all the aforementioned objectives. This consistency suggests that localized image-generation artifacts are unlikely to be the primary factor behind the observed results and instead points to an inherent characteristic of the diffusion architecture.

GAN-based translation methods like CUT prioritize high-frequency pixel alignment and perceptual fidelity, frequently at the expense of altering spatial layouts [27]. Conversely, ControlNet promotes structural consistency through the use of zero-convolution layers [33]. Structurally, as implemented in the presented pipeline, the control sub-network processes the input conditional maps through a sequence of convolutional residual blocks coupled with Multihead Self-Attention and Cross-Attention layers. The Self-Attention modules capture long-range spatial dependencies across the source layout, helping to preserve topological continuity (e.g., road connectivity or urban block distribution). Meanwhile, the Cross-Attention layers directly guide the latent denoising process according to the geometric constraints provided by the conditioning tiles. Consequently, even when the generated imagery exhibits imperfect surface textures, the underlying geometric topology and spatial continuity tend to be preserved. For a downstream segmentation architecture, these highly stable structural cues seem to provide a cleaner, more reliable training signal than visually pristine but geometrically distorted GAN outputs.

Moreover, the results highlight the potential of synthetic data as a complementary source of training samples. When artificial images were combined with authentic satellite scenes, the segmentation models achieved the best performance observed in this study. This finding aligns with previous works highlighting the importance of synthetic data to mitigate the scarcity of annotated Earth observation datasets [6,8,10].

This work also acknowledges several limitations that open the door to future research. Firstly, the study was underpinned by a single dataset, composed of diverse maps associated with scenes covering different regions of Spain. With this data, the adapted models were able to synthesize large-scale entities such as rivers, buildings, roads, seas, or parks. Nevertheless, the generation of finer details such as cars or ships remains a significant challenge for addressing more intricate computer vision detection tasks.

Furthermore, this investigation was centered on converting maps to satellite images resembling the characteristics of a specific sensor. Extending this milestone to make possible the translation from one source sensor to any other desired sensor would be highly relevant. This research path perfectly aligns with the goal of reducing the real imagery requirements while retaining (or even improving) the performance of neural networks through data augmentation. Achieving these objectives would represent a substantial advancement toward on-board EO processing without acquiring numerous real examples beforehand, considering their inherent scarcity and high acquisition cost.

In addition, the programmatic creation of artificial satellite scenes through map tiles offers a multitude of unexplored possibilities. For instance, this strategy would enable the creation of specialized datasets tailored to a specific biome or region by using custom maps (either handmade or automatically generated) that follow the contrastive color patterns on which the generative architectures were trained.

Finally, future work is required to assess the effectiveness of larger-scale proposals than the ones implemented in this research. This would allow measuring the knowledge transfer to real-world tasks from synthetic outputs provided by models such as Imagen [31] or DALL-E 2 [32], deployed without the computational restrictions inherent to low-power, edge device hardware.

## 6. Conclusions

This work sought to explore the feasibility of knowledge transfer from synthetic imagery to Earth Observation (EO) tasks. Specifically, the objective was to investigate that the strategic hybridization of authentic satellite data with synthetic samples produced by generative AI models provides an effective data augmentation strategy, effectively alleviating the scarcity of scenes with specific sensor characteristics.

To achieve this, this study compared multiple state-of-the-art methods, including Pix2Pix, CUT, and the ControlNet strategy. These architectures represent a spectrum of sophisticated approaches, ranging from conditional and contrastive GANs to cutting-edge LDMs that hybridize DDPMs with VAEs and advanced prediction control mechanisms.

The experimental setup involved training these networks under identical conditions to synthesize satellite scenes from regular map tiles. Due to the lack of suitable public datasets, a custom dataset featuring diverse regions of Spain was curated. By assigning high-contrast colors to map entities, the learning process was streamlined across all generative architectures. Subsequently, to objectively assess these models beyond visual inspection, a downstream semantic segmentation framework was deployed: multiple U-Net instances were optimized strictly on the synthetic collections produced by each generator and subsequently validated against a test set composed exclusively of authentic satellite images, focusing on the detection of buildings, roads, and waterbodies.

The obtained results indicate that knowledge transfer from artificial imagery to EO tasks is not only feasible but can provide advantages for neural network training. In addition, this research reveals a nuanced relationship between perceptual realism and functional utility; while some architectures produce more visually convincing scenes, others can still provide representative features that contribute to competitive downstream task accuracy. This finding underscores the importance of task-oriented evaluation metrics over purely visual assessments in data generation.

Experimental results showed that integrating synthetic samples as a data augmentation strategy improved the performance, raising Dice scores to 54.1% (up from 53.2% real-only) for buildings, 38.6% (up from 36.3%) for roads, and 46.5% (up from 42.4%) for waterbodies. Consequently, this hybrid approach appears to be a promising and cost-effective strategy for alleviating the scarcity of annotated real-world satellite imagery. Future work will explore the generalizability of this framework across different spatial resolutions and alternative EO modalities.

## Figures and Tables

**Table 1:** Correspondence between the colors applied to the dataset’s maps and their target categories.

Color	Land-Cover Class
Red	Buildings, factories, and any other human construction
Yellow	Roads, bridges, tunnels, and highways
Pink	Pedestrian or cycling paths
Blue	Lakes, rivers, the sea, and any other water surfaces
Green	Parks, forests, and crops
Brown	Mountains and land
White	Background / Other surfaces

**Table 2:** Summary of the primary hyperparameter configurations utilized across the evaluated models.

Model	Component	Learning Rate	Optimizer	Scheduler	Loss Function
Pix2Pix	Discriminator	$2\times 10^{-4}$	Adam	-	${\mathrm{BCE}}_{I}$
	Generator	$2\times 10^{-4}$	Adam	ReduceLROnPlateau	${\mathrm{BCE}}_{I}$ + ${\mathrm{MAE}}_{R}$
CUT	Discriminator	$2\times 10^{-4}$	Adam	-	${\mathrm{MSE}}_{I}$
	Generator	$2\times 10^{-4}$	Adam	-	${\mathrm{MSE}}_{I}$ + NCE
ControlNet	VAE Discriminator	$1\times 10^{-5}$	Adam	-	${\mathrm{MSE}}_{I}$
	VAE Generator	$1\times 10^{-5}$	Adam	-	${\mathrm{MSE}}_{R}$
	LDM	$2.5\times 10^{-5}$	Adam	MultiStepLR	${\mathrm{MSE}}_{R}$
	Complete network	$1\times 10^{-5}$	Adam	MultiStepLR	${\mathrm{MSE}}_{R}$
U-Net	-	$1\times 10^{-5}$	RMSProp	ReduceLROnPlateau	${\mathrm{BCE}}_{S}$

**Table 3:** Summary of the U-Net’s segmentation performance on the authentic imagery test set.

	TRAINING AND VALIDATION IMAGES	COMPLETE TEST SET		
		PRECISION	RECALL	DICE SCORE
**BUILDINGS**	Real	**0.607**	0.575	0.532
	Pix2Pix	0.458	0.052	0.032
	CUT Unpaired	0.280	0.354	0.257
	CUT Paired	0.418	0.317	0.286
	ControlNet	0.375	**0.735**	0.444
	Real + CUT Paired	0.541	0.526	0.484
	Real + ControlNet	0.559	0.630	**0.541**
**ROADS**	Real	0.465	0.342	0.363
	Pix2Pix	**1.000** ^1^	0.020	0.020
	CUT Unpaired	0.326	0.113	0.141
	CUT Paired	0.405	0.186	0.225
	ControlNet	0.533	0.132	0.167
	Real + CUT Paired	0.670	0.299	0.380
	Real + ControlNet	0.454	**0.381**	**0.386**
**WATER**	Real	0.558	0.795	0.424
	Pix2Pix	0.047	**0.853**	0.043
	CUT Unpaired	0.520	0.774	0.400
	CUT Paired	0.427	0.790	0.315
	ControlNet	0.312	0.787	0.226
	Real + CUT Paired	0.524	0.808	0.420
	Real + ControlNet	**0.599**	0.800	**0.465**

^1^ This value reflects a failure mode where the segmentation model predicted all pixels as background, resulting in zero false positives but failing to learn useful features.

**Table 4:** Performance comparison of the U-Net on urban city centres and the rest of authentic scenes.

	TRAINING AND VALIDATION IMAGES	URBAN CITY CENTRES			REST OF SCENES		
		PRECISION	RECALL	DICE SCORE	PRECISION	RECALL	DICE SCORE
**BUILDINGS**	Real	**0.656**	0.654	**0.634**	**0.531**	0.454	0.375
	Pix2Pix	0.433	0.005	0.008	0.495	0.124	0.068
	CUT Unpaired	0.382	0.367	0.348	0.123	0.334	0.116
	CUT Paired	0.540	0.321	0.370	0.230	0.311	0.158
	ControlNet	0.460	**0.771**	0.551	0.243	**0.680**	0.279
	Real + CUT Paired	0.634	0.576	0.587	0.398	0.450	0.326
	Real + ControlNet	0.642	0.659	**0.632**	0.431	0.586	**0.401**
**ROADS**	Real	0.470	0.333	0.376	0.456	0.357	0.343
	Pix2Pix	**1.000** ^1^	0.000	0.000	**1.000** ^1^	0.050	0.050
	CUT Unpaired	0.341	0.095	0.139	0.303	0.140	0.144
	CUT Paired	0.431	0.180	0.239	0.364	0.196	0.203
	ControlNet	0.501	0.124	0.169	0.583	0.145	0.156
	Real + CUT Paired	0.702	0.294	**0.398**	0.621	0.307	0.352
	Real + ControlNet	0.457	**0.368**	0.389	0.448	**0.400 **	**0.382**
**WATER**	Real	0.514	**0.858**	0.422	0.627	0.698	**0.426**
	Pix2Pix	0.013	0.917	0.017	0.100	**0.755 **	0.084
	CUT Unpaired	0.468	0.844	0.413	0.599	0.665	0.380
	CUT Paired	0.373	0.858	0.309	0.510	0.686	0.325
	ControlNet	0.269	0.852	0.219	0.378	0.688	0.236
	Real + CUT Paired	0.501	0.866	0.430	0.560	0.718	0.405
	Real + ControlNet	**0.570**	**0.858**	**0.492**	**0.643**	0.712	**0.424 **

^1^ This value reflects a failure mode where the segmentation model predicted all pixels as background, resulting in zero false positives but failing to learn useful features.

## Data Availability

The data supporting the conclusions of this article will be made available by the authors on request.
